# Extrapulmonary Tuberculosis Mimicking an Iliac Bone Lytic Lesion: A Case Report

**DOI:** 10.7759/cureus.85216

**Published:** 2025-06-02

**Authors:** Mohammed Mushabbab Alobud, Sami Amer M Alqarni, Bandar Saeed Alqahtani, Thamer Yahya Alasiri, Ali Abdullah Alshehri

**Affiliations:** 1 Orthopaedic Surgery, Armed Forces Hospital Southern Region, Khamis Mushait, SAU

**Keywords:** extrapulmonary tb, granulomatous osteomyelitis, iliac bone, lytic bone lesion, tuberculous osteomyelitis

## Abstract

Lytic bone lesions pose significant diagnostic challenges due to their varied causes, ranging from malignancies to infections and benign conditions. Tuberculous osteomyelitis, though rare in non-endemic regions, remains an important consideration, particularly in patients from high-burden areas. A 35-year-old Sudanese male with a family history of tuberculosis (TB) presented with chronic left iliac pain that did not respond to nonsteroidal anti-inflammatory drugs (NSAIDs). Imaging revealed an expansile lytic lesion with cortical breaching, initially raising suspicion for malignancy. However, a biopsy showed necrotizing granulomatous osteomyelitis, though cultures - including those for *Mycobacterium tuberculosis - *were negative. Despite the lack of microbiological confirmation, the patient showed clinical and radiological improvement after starting empirical anti-TB therapy. This case highlights the need to consider tuberculous osteomyelitis in the differential diagnosis of lytic bone lesions, even in the absence of positive cultures, especially in individuals from endemic regions. Histopathological evidence of granulomas and a positive response to anti-TB therapy can support the diagnosis when microbiological tests are inconclusive. Greater awareness of this possibility is essential to prevent delays in treatment and unnecessary invasive procedures.

## Introduction

Lytic bone lesions can result from various factors, including benign, malignant, or infectious origins. An extensive differential diagnosis and careful observation are essential for accurate diagnosis and effective management. Many diagnostic tests that differentiate these conditions require biopsy; thus, it is important to recognize the potential causes of such lesions and proactively order the relevant diagnostic tests to avoid unnecessary procedures or treatments.

Bone and joint tuberculosis (TB) currently represents 2.2%-4.7% of all TB cases in Europe and the USA, and approximately 10%-15% of extrapulmonary tuberculosis (EPTB) cases in developing countries, particularly in Asia [1].

Despite its infrequency, bone TB significantly contributes to lytic bone lesions. The diagnosis is often missed, resulting in inadequate and potentially harmful treatments. This report outlines a case of a patient who presented with a lytic bone lesion, where biopsy results were positive for granulomatous osteomyelitis and subsequent culture was negative for the presence of *Mycobacterium tuberculosis*; yet, the patient reacted favorably to anti-TB treatment.

## Case presentation

A 35-year-old Sudanese male from a TB-endemic area, with a positive family history of tuberculosis and no personal history of the disease, presented with a two-year history of pain in the left upper thigh and gluteal region, previously managed with nonsteroidal anti-inflammatory drugs (NSAIDs). He reported a history of trauma in 2021 but denied experiencing fever, weight loss, or other systemic symptoms. He was referred to our Musculoskeletal Oncology Unit from a peripheral hospital in September 2023.

Physical examination revealed the patient to be in generally good health, with normal vital signs. No mass was detected in the left gluteal region. The left hip's range of motion was normal, with mild pain, and other examination results were unremarkable.

**Figure 1:**
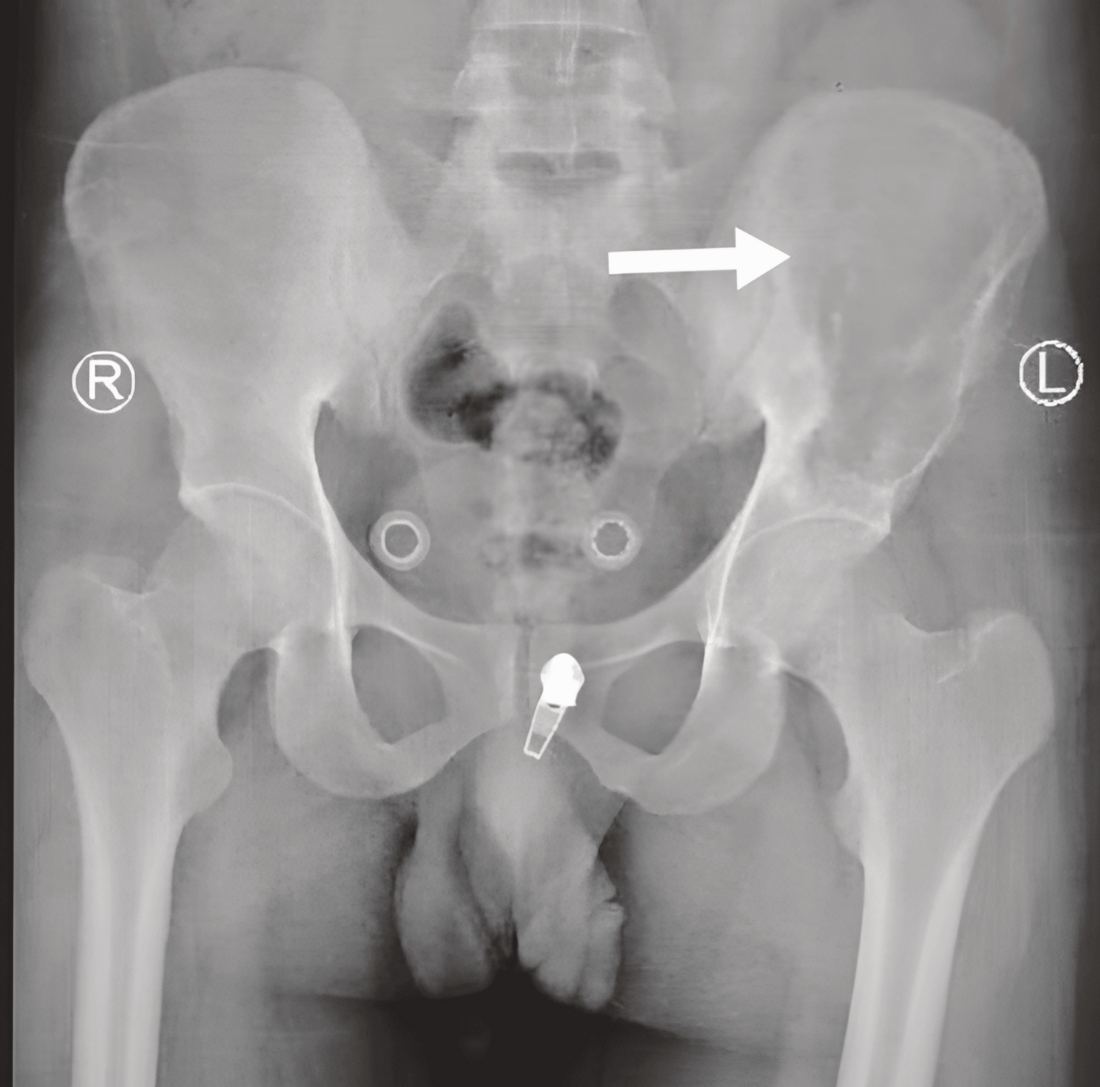
Plain X-ray of pelvis demonstrate left iliac bone lytic lesion (arrow)

**Figure 2:**
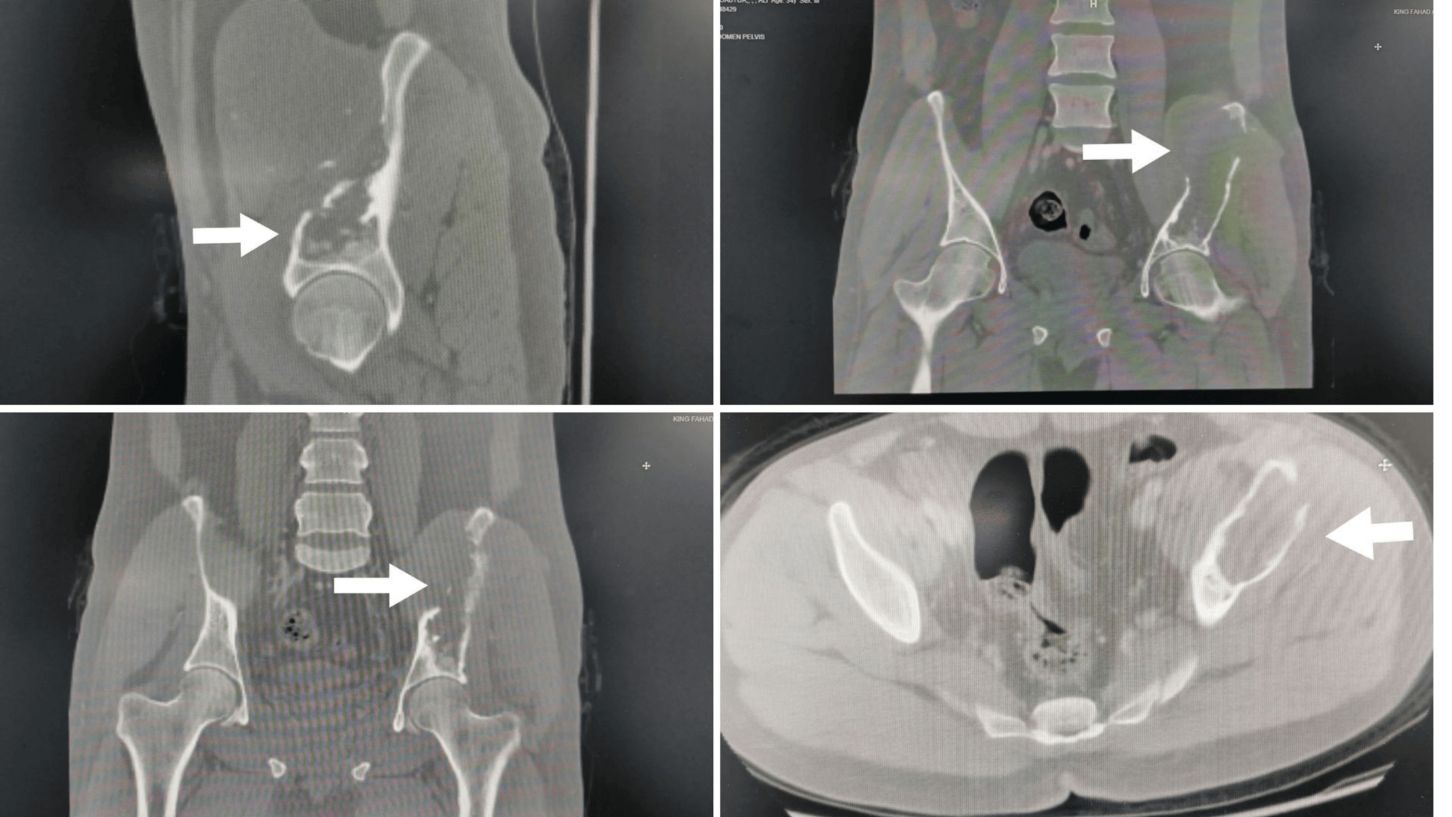
CT pelvis coronal, sagittal, and axial (bone window) demonstrate left iliac bone lytic lesion with evident of cortical breaching (arrows) CT, computed tomography

**Figure 3:**
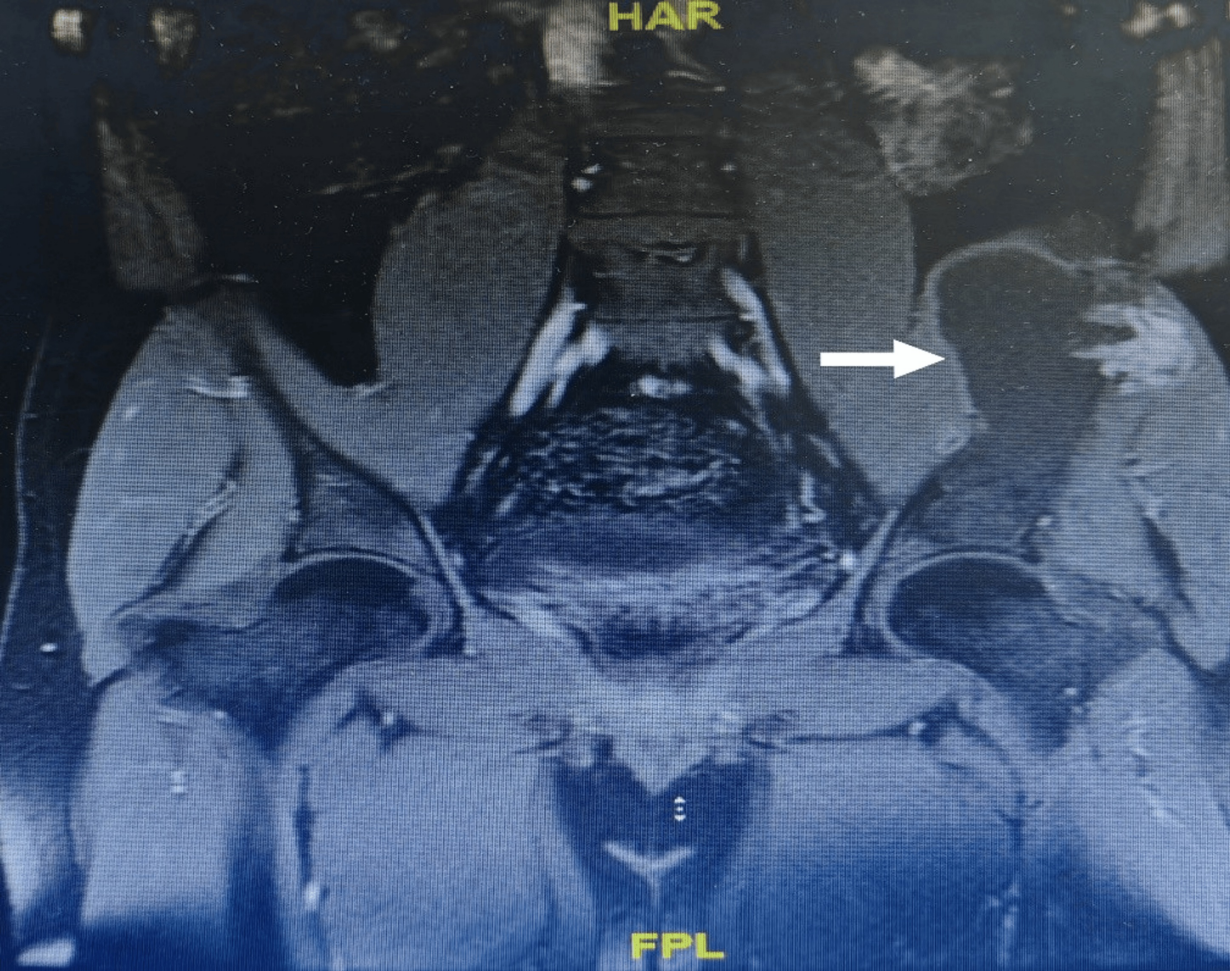
MRI pelvis with intravenous contrast demonstrate left iliac bone lytic lesion with evident of cortical breaching MRI, magnetic resonance imaging

Laboratory tests indicated a normal white blood cell (WBC) count, erythrocyte sedimentation rate (ESR), and C-reactive protein (CRP) (Table 1). A plain radiograph of the pelvis showed a lytic lesion in the left iliac bone (Figure 1). The chest X-ray was unremarkable for TB. Computed tomography (CT) of the pelvis (Figure 2) and magnetic resonance imaging (MRI) of the pelvis with intravenous (IV) contrast (Figure 3) revealed a complex expansile lytic lesion in the left iliac bone with evident cortical breaching, raising suspicion for a neoplastic process. Subsequently, CT scans of the chest, abdomen, and pelvis were ordered for staging, which fortunately showed no evidence of metastasis. However, a Tru-cut biopsy yielded negative results for neoplasm, instead revealing necrotizing epithelioid cell granulomas and a giant cell histiocytic reaction, negative for specific microorganisms. The morphologic features are those of a necrotizing granulomatous reaction. Although no specific microorganisms were seen on routine and special stains (including AFB stains for *M. tuberculosis* and PAS/D for fungal elements), an infectious etiology (including *M. tuberculosis*) cannot be ruled out. The culture from the lesion did not reveal any organisms, including TB.

**Table 1:** Laboratory results show normal WBC and inflammatory markers WBC, white blood cell; CRP, C-reactive protein; ESR, erythrocyte sedimentation rate

Test	Result	Reference Range
WBC	6.31 × 10^9^/L	4.5-13.5 × 10^9^/L
CRP	2.7 mg/L	<5 mg/L
ESR	2 mm/h	<10 mm/h

**Figure 4:**
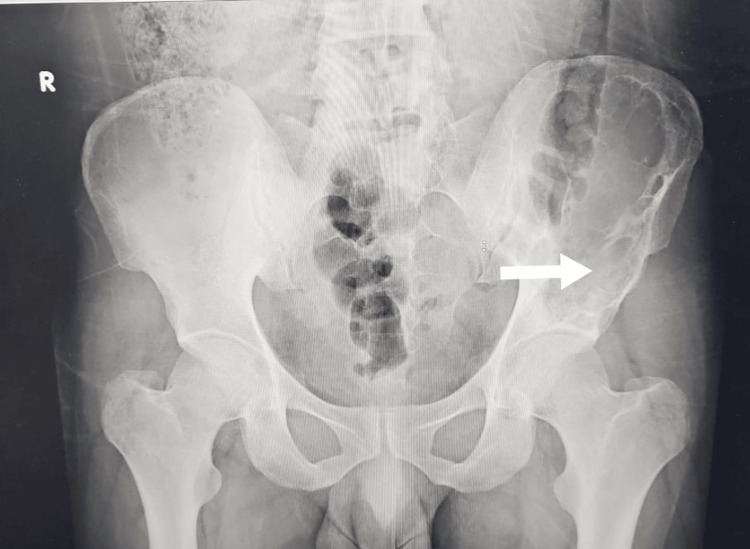
Follow up plain X-ray of pelvis shows the improvement of bone quality in the left iliac bone (arrow)

**Figure 5:**
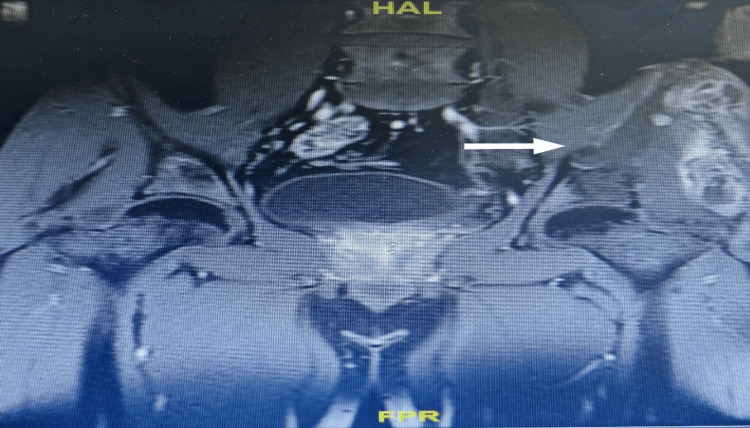
Follow up MRI pelvis with intravenous contrast demonstrate left iliac bone lytic lesion regression (arrow) MRI, magnetic resonance imaging

Given the pathology and microbiology findings, the patient was diagnosed with tuberculous osteomyelitis of the left iliac bone. Treatment commenced with isoniazid (300 mg once daily), rifampin (600 mg once daily), pyrazinamide (1500 mg once daily), ethambutol (1200 mg once daily), and pyridoxine (40 mg once daily) for 60 days, then isoniazid (300 mg once daily), rifampin (600 mg once daily), and pyridoxine (40 mg once daily) for one year, administered under directly observed therapy. The patient currently shows signs of clinical and radiological improvement (Figures 4-5).

## Discussion

Isolated iliac bone TB is a rare condition, representing less than 1% of skeletal TB cases [2]. The rare localization of the disease, its capacity to resemble other pathological conditions, and the insufficient awareness among physicians complicate early diagnosis [2,3]. Hematogenous spread from a primary infection is the typical route for tuberculous osteomyelitis [2]. This primary infection may be active or dormant, clinically evident or hidden, and can originate in the lungs or other internal organs [4].

The correlation between skeletal TB and pulmonary manifestations of the disease is approximately 50% [5,6]. Consequently, there may be a reduction in the consideration of TB as a diagnostic possibility in patients presenting with negative chest X-ray results. The chest X-ray was normal, leading to the exclusion of TB in the initial clinical differential diagnosis. Recent cases of iliac TB predominantly involve immunocompromised patients [2]. Our patient, who was not immunocompromised, received initial treatment with NSAIDs. As a result, his symptoms were masked, delaying the diagnosis. Currently, global migration has led to the emergence of such cases outside endemic regions, resulting in complex diagnostic challenges. The patient's origin led us to prioritize the radiological diagnosis of TB as the primary consideration.

The radiological manifestations of skeletal TB differ based on the disease stage. Initial lesions may be overlooked, as X-rays typically show normal bone, with soft tissue swelling often being the sole indication of abnormality [7,8]. Localized osteopenia may subsequently develop, accompanied by the formation of destructive bony foci and minimal surrounding sclerosis. The healing process is characterized by gradually eliminating the destructive focus and developing marginal sclerosis [9]. A lytic area, characterized by a hazy, irregular, soft shadow at the center and accompanied by minimal surrounding sclerosis, may be observed in cancellous bones such as the iliac bone [2]. This finding, along with the inadequate response to NSAIDs, necessitated MRI imaging. The MRI yielded more accurate and detailed information regarding the lesion. The site, size, and lytic characteristics of the bony component of the lesion were accurately depicted.

Histological evaluation is essential for accurate diagnosis. Biopsies of the bone lesions, synovial tissues, or soft masses are crucial for clarifying the diagnosis. A study by Enache et al., involving 19 cases of osteoarticular TB, found that biopsy confirmed the diagnosis in all instances, displaying epithelioid granulomas and caseous necrosis [10]. In another study by Muangchan et al., biopsy confirmed the diagnosis in 46.5% of cases [11]. The gold standard for diagnosis remains the culture of *M. tuberculosis* from the affected bone tissue. In the current cases, patients presented with granulomatous osteomyelitis upon open biopsy, with cultures confirming *M. tuberculosis*, leading to the diagnosis of tuberculous osteomyelitis.

The primary treatment approach for tuberculous osteomyelitis involves anti-TB drug therapy. Surgical interventions, such as joint fusion or replacement, may be required in advanced cases with significant joint immobility.

## Conclusions

This report highlights the diagnostic complexities of isolated iliac bone TB, a rare extrapulmonary TB manifestation. The patient presented with lytic bone lesions without pulmonary involvement or immunocompromise, and initial microbiological cultures were negative. Diagnosis relied on clinical suspicion, histopathological evidence of granulomatous osteomyelitis, and a favorable response to empiric anti-TB therapy. The case underscores three critical considerations: first, TB should remain high on the differential for lytic bone lesions in patients from endemic regions, even in the absence of classic risk factors or systemic symptoms. Second, microbiological confirmation - though ideal - may not always be achievable; in such cases, histopathological findings of granulomas and therapeutic response can support the diagnosis. Third, multidisciplinary collaboration among radiologists, pathologists, and infectious disease specialists is essential to distinguish tuberculous osteomyelitis from malignancies or pyogenic infections, preventing diagnostic delays or inappropriate interventions.

Future research must prioritize advancing diagnostic tools, particularly polymerase chain reaction (PCR)-based assays, to improve the detection of culture-negative tuberculous osteomyelitis. This case reinforces that early recognition and prompt initiation of anti-TB therapy are crucial to mitigate complications like structural damage or chronic disability. Clinicians should maintain heightened awareness of TB’s atypical presentations, especially in endemic areas, while advocating for improved diagnostic frameworks. By integrating clinical judgment with evolving technologies, healthcare teams can optimize outcomes for these diagnostically challenging cases, ultimately reducing the morbidity associated with overlooked or misdiagnosed extrapulmonary TB.
